# Investigation on Microstructures and Mechanical Properties of the Hypoeutectic Al-10Si-0.8Fe-*X*Er Alloy

**DOI:** 10.1155/2020/9147871

**Published:** 2020-04-29

**Authors:** Peng Tang, Yiyuan Liu, Yanjun Zhao, Zhiliu Hu, Huachun Wang, Linxin Peng, Songyun Deng, Kui Huang

**Affiliations:** ^1^School of Resources, Environment and Materials, Guangxi University, Nanning, China 530004; ^2^Guangxi Key Laboratory of Processing for Non-ferrous Metal and Featured Materials, Guangxi University, Nanning, China 530004; ^3^Guangxi Alnan Institute of Aerospace Transit Aluminum Alloy and Application, Nanning, China 530031; ^4^School of Civil Engineering and Architecture, Guangxi University, Nanning, China 530004

## Abstract

In this paper, the effect of Er addition (0.2, 0.5, 0.65, 0.8, 1.0, and 1.5 wt. %) on the microstructure evolution and tensile properties of as-cast hypereutectic Al-10Si-0.8Fe alloy was investigated. The phases and their morphologies in these alloys were identified by XRD and SEM equipped with EDX with the help of metallographic analysis techniques; the length of the secondary phase (LSP) and secondary dendrite arm spacing (SDAS) of *α*-Al grain were quantified. The results indicated that the second phases (primary Si, eutectic Si, and iron-rich phases) and *α*-Al grain were significantly refined when the addition of Er increased from 0 to 0.8 wt. %. The mean LSP and SADS values were decreased to a minimum value when the Er addition reached 0.8 wt. %. However, the second phases and *α*-Al grain became coarser when the level of Er increased more than 0.8 wt. %. The analysis of XRD shows that Er mainly exists in the form of Er_2_Si compound. The microstructure modification also has a significant effect on the mechanical properties of the alloy. The yield strength (YS), ultimate tensile strength (UTS), and elongation (EL) increase from 52.86 MPa, 163.84 MPa, and 3.45% to 71.01 MPa, 163.84 MPa, and 5.65%, respectively. From the fracture surface, the promotions of mechanical properties are due to the dispersion and pinning reinforcement caused by the Er_2_Si phase.

## 1. Introduction

Al-Si alloys have low density, high strength, and excellent mechanical properties, which are widely used in the aerospace industry, the automotive industry, the construction industry, etc. [1–3]. With the development of science and technology, the performance requirements of cast Al alloy materials in various industries are constantly increasing. There are many ways to improve the performance of cast Al alloys, among which grain refinement is an efficient method [4, 5]. The commonly used refiners are Ce, La, Mn, and Ti [6–9], although research on refiners has achieved some results. But unfortunately, no efficient and cheap grain refiner has appeared. Therefore, better refinement effect and smaller cost consumption are the topics of continuous research by domestic.

In the processing of Al-Si alloys, a certain amount of Fe element is inevitably added. In order to simulate the actual use situation as much as possible, 0.8 wt% 在 element will be added in the experiment. Before the addition of a refiner, the second phase in Al-Si alloys is a bulk or plate-like Si phase, and long needle-like *β*-Fe phase appears when the Fe content increased [10–14]. The formation of these phases has an impact on the mechanical properties of aluminum alloys and reduces various mechanical properties. According to Qian et al.'s research [15], Er has a better regulation effect on the secondary phase in aluminum alloys. And Tantiwaitayaphan et al. [16] also reported that the addition of Er can reduce the degree of subcooling and cause the morphology of eutectic silicon to change. These research show that the rare earth element Er can improve the morphology of the secondary phase and improve the mechanical properties of the aluminum alloys [17–21], but it has not been proposed which kind of particles the Er element will form with the Al-Si alloys to affect the morphology of the secondary phase.

In this experiment, Al-10Si-0.8Fe will be taken as an object. By increasing the content of the Er element, the effect of Er on the microstructure morphology, mechanical properties, and fracture behavior of the Al-10Si-0.8Fe alloy will be explored. The size of grains, the secondary phases, and the properties between secondary phases and the matrix would prominently impact the strength of these materials. And the second phases include primary silicon, eutectic silicon, and iron-rich phases. Therefore, we analyzed the statistical results of the secondary dendritic arm spacing (SDAS) and the length of second phase(LSP). By means of X-ray diffraction (XRD), scanning electron microscope (SEM)/energy-dispersive X-ray spectroscopy (EDX), and tensile fracture morphology, the principle and mechanism of adding the Er element to Al-Si-Fe alloy to enhance its properties were studied.

## 2. Experimental Materials and Methods

These hypoeutectic Al-10Si-0.8Fe-*X*Er (*X* = 0.0, 0.2, 0.5, 0.65, 0.8, 1.0, and 1.5 wt.%) alloys were developed using commercially pure Al (99.0 wt. %), Al-12Si master alloy, Al-75Fe master alloy, and Al-3Er master alloy through gravity casting method. There are also surface coatings (ZnO : Na_2_SiO_3_ : H_2_O = 2 : 1 : 7), covering agents (NaCl : KCl = 1 : 1), refining agents C_2_Cl_6_, etc. The experiment was performed in a crucible resistance furnace, and the mass of the alloy smelted in each furnace was 600 g. The chemical compositions of the experimental alloys are listed in Table 1 (measured by the direct-reading spectrometer, SPECTROLAB/M11, Germany). The specific process is that the alloy Al-12Si and pure aluminum block are melted at 780°C, and the prepared iron agent is added, and the stirring is continued for 2 minutes to make the crucible composition more uniform and keep the temperature for 25 minutes. After raising the temperature to 800 ± 5°C, add an appropriate amount of Al-3Er intermediate alloy, stir and keep it for 25 min. The melt temperature is reduced to 720 ± 5°C, and the refining agent is added for deaeration after standing for 5 minutes. Finally, the slag pouring is performed, and the pouring temperature is 750 ± 5°C. Samples for metallurgical analysis and tensile testing were machined out from these cast ingots and cooled down at room temperature.

After the sample is prepared, the test sample is cut to obtain grinding, polishing, and corrosion treatment with HF solution (composed of 0.5 ml hydrofluoric acid and 100 ml H_2_O) in order to better observe the metallographic phase. The metallographic analysis was conducted by the optical microscope (Zeiss/Observer. A1, Germany). The mean values of LSP and SDAS were evaluated using Nano-measurement software and statistical methods. At least 50 statistical samples were also randomly determined in each of the five view fields. Similar with the relevant literatures, the secondary dendrite arm spacing (SDAS) value and lengths of the second phases (LSP) were measured for investigating the effect of the added elements in these five view fields:

(1) $$\begin{array}{c} \text{Mean }{\text{L}}_{\text{i}}=\frac{1}{m}\overset{m}{\underset{j=1}{\sum }}{\left(\frac{1}{n}\overset{n}{\underset{i=1}{\sum }}L_{i}\right)}_{j}, \end{array}$$

where *L*_*i*_ is the LSP and SDAS of an arbitrary phase in the microstructure, *n* is the number of particles measured in a view field, and *m* is the number of the fields for quantification. In this case, the *n* value is determined as 50 and *m* is 5.

Meanwhile, these phases were observed by SEM (Hitachi TM4000Plus) coupled with EDX (IXRF 5500, USA) in this experiment, and the phase composition of the bulk sample was analyzed by XRD. Then, a universal testing machine is used to perform the tensile test to obtain the yield strength (YS), ultimate tensile strength (UTS), and elongation (EL) which were obtained at a strain rate of 1 mm/min. According to the ASTM E8M-04 standard, five tensile testing samples were machined out for each alloy. The fracture surfaces of these specimens were further investigated via the SEM.

## 3. Results

### 3.1. Evaluation Microstructure of Al-10Si-0.8Fe-*X*Er Alloys

**Figure 1:**
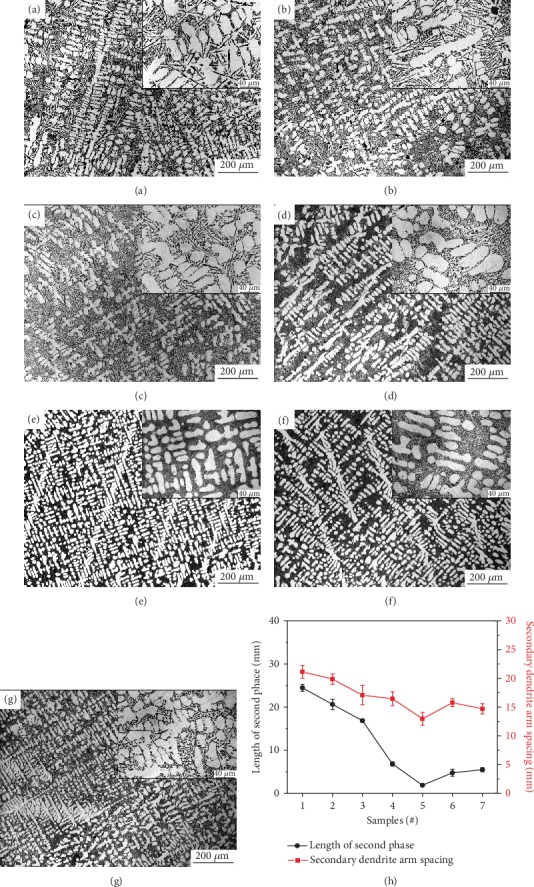
Optical micrographs of Al-10Si-0.8Fe alloys with different Er additions: (a) unmodified, (b) 0.20 wt.%, (c) 0.50 wt.%, (d) 0.65 wt.%, (e) 0.80 wt.%, (f) 1.00 wt.%, (g) 1.50 wt.%, and (h) statistical curve.

The optical micrographs of Al-10Si-0.8Fe-*X*Er (*X* = 0.0, 0.2, 0.5, 0.65, 1.0, and 1.5) alloys are shown in Figure 1. In Figure 1(a), the Al-10Si-0.8Fe alloy contains needle-like second phase distributed around the dendritic *α*-Al, and the secondary dendrite arm spacing is relatively large. After adding the rare earth Er, the second phases were significantly refined from coarse polygonal and star-like shape to a fine block with smooth edges and corners when the addition of Er increased from 0 to 0.8%.When Er was added at 0.8 wt, the second phase had the best metamorphic effect and was the densest, and the second phase was refined into small particles, as shown in Figure 1(e). However, the second phases became coarser when the level of rare earth Er is more than 0.8%.

Figure 1(upper right corner) shows higher magnification metallographic images of these alloys. The Al-10Si-0.8Fe alloy contains gray, coarse needle-like eutectic silicon phase which is distributed around the *α*-Al phase, and the segregation is relatively serious; there are also a small number of polygonal bulky primary silicon phases in the structure. Most of the iron-rich phases are light gray, skeletal, or Chinese characters. They are entangled with eutectic silicon and adhere to the periphery of the *α*-Al phase. When Er is 0.8%, the *α*-Al dendrite grain boundaries have almost no edges, the shape becomes round and regular, and the structure becomes dense. The eutectic silicon distributed in the *α*-Al grain boundary is almost completely refined, and changes from a coarse needle-like shape to a dispersedly distributed fine particle or dot network.

The results in Figure 1(h)) indicated the eutectic silicon modification and the *α*-Al refinement effect of Er in the Al-10Si-0.8Fe alloy. When there is no Er element, the mean LSP and the SDAS values are 24.47 *μ*m and 37.47 *μ*m, respectively. As the amount of Er added increases, the mean LSP and SDAS values decrease rapidly. When the Er addition of Al-10Si-Fe alloy is 0.8%, the effect of refine and modification is the most obvious. The mean LSP and SDAS values reach the minimum value of 1.87 *μ*m and 12.97 *μ*m, respectively. When the amount of Er added continued to raise, the mean LSP gradually raised. Meanwhile, the value of SDAS gradually rose. After that, the SDAS values download again. Generally speaking, the SDAS value still showed an upward trend.

**Figure 2:**
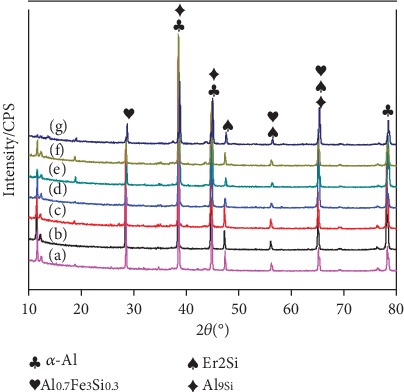
XRD patterns of alloys with different addition amounts of Er.

The XRD patterns of the rare earth element Er with different addition amounts are shown in Figure 2. The Er element will react with the silicon in the aluminum alloy to form an Er_2_Si phase. At the same time, Al_9_Si, Al_0.7_Fe_3_Si_0.3_ and other phases also exist in the aluminum alloy.

**Figure 3:**
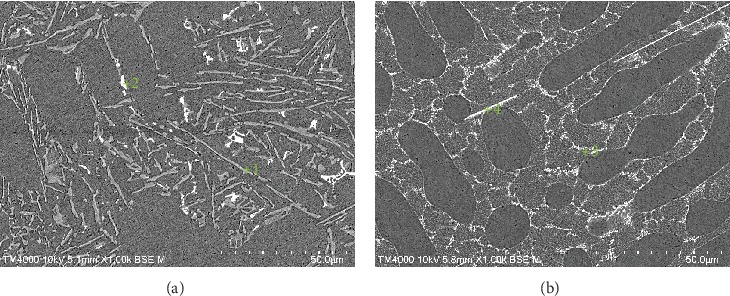
SEM morphology of alloys with different additions of Er: (a) 0.20 wt.% and (b) 0.80 wt.%.

The SEM with 0.2% and 1.0% Er are shown in Figure 3, and EDX are shown in Table 2. From Figure 3(a), the eutectic silicon in these alloys is mainly long and massive. According to the XRD and EDX, the gray needle phase at point 1 is presumed to be the Al-Si phase, and the white block at point 2 is the Al_0.7_Fe_3_Si_0.3_ phase. From Figure 1, the alloy has been finely refined, and the eutectic silicon mainly appears in the form of short rods, particles, and thin strips. According to XRD and EDX, it can be inferred that the white particles in point 3 are the Er_2_Si phase, and the white thin strips in point 4 are the AlSiEr phase. This shows that with the increase of Er content, the Er element starts to react with Al and Si to generate particles and thin phase. The addition of the Er element changed the morphology of the silicon phase and prevented its growth so that the second phase was refined.

### 3.2. Tensile Properties of the Al-10Si-0.8Fe-*X*Er Alloy

Figure 5 is a graph of the YS, UTS, and EL% of the Al-10Si-0.8Fe-*X*Er alloys. From Figure 5, the addition of Er improves the YS of the Al-10Si-0.8Fe alloy. The YS of the Al-10Si-0.8Fe alloy is 52.86 MPa. With the increase of the Er content, the YS of these alloys continues to increase. When the Er content increases from 0.5% to 0.65%, the YS of the alloy increases the most, 13.4%.When the content is 0.8%, the YS of the alloy is 71.01 MPa, which reaches the maximum value, which is about 34.3% higher than that of the Al-10Si-0.8Fe alloy. Once Er content is more than 0.8 wt.%, the yield strength of the alloy decreased significantly. The addition of Er element refines the eutectic silicon into fine particles, and the structure is uniform and dense. According to the Hall-Petch formula

(2) $$\begin{array}{c} \sigma =\sigma_{0}+Kd^{-1/2}, \end{array}$$

where *σ* is the yield strength, MPa; *σ*_0_ is the yield strength of the single crystal, MPa; *d* is the grain size; and *K* is a constant.

With the refinement of grains, the interfacial area of the grains increases per unit volume and the grain boundaries hinder the movement of dislocations. The deformation caused by the stress during the stretching process can be dispersed into more grains. A great quantity of grain boundaries effectively hinders the movement of dislocations, and the dislocations continue to accumulate. Difficulty increases, and the yield strength of alloy materials in macroperformance increases. The experimental results show that when Er content is 0.8%, the grain size of the alloy structure reaches the minimum, and the yield strength reaches the maximum.

From Figure 5, the Er element also improves the UTS and %EL of the Al-10Si-0.8Fe alloy. When Er content is 0.8%, UTS reaches the maximum value of 213.31 MPa, increasing by 30.2%. When the content of Er exceeds 0.8%, UTS decreases, which may be due to the high content of Er and the large amount of long-needle iron-rich phase precipitation. Generally, with the increase of Er, the UTS of these alloys increases first and then decreases. When the content of Er is 0.8%, the UTS of these alloys are the highest. The %EL after fracture of the original alloy sample was 3.43%. When the Er content was 0.2%, eutectic silicon was refined and the %EL at break of the alloy increased slightly. When the content of Er increased to 0.5% and 0.65%, the length of the iron-rich phase in the tissue became longer, the morphology was mostly acicular, and the elongation rate decreased. The grain refining effect is best when Er content is 0.8%, the length of the iron-rich phase becomes shorter, the grain refining improves the deformation resistance of the alloy, and the elongation of the alloy reaches the maximum value. The maximum value was 5.49%, increasing by 60%.

**Figure 4:**
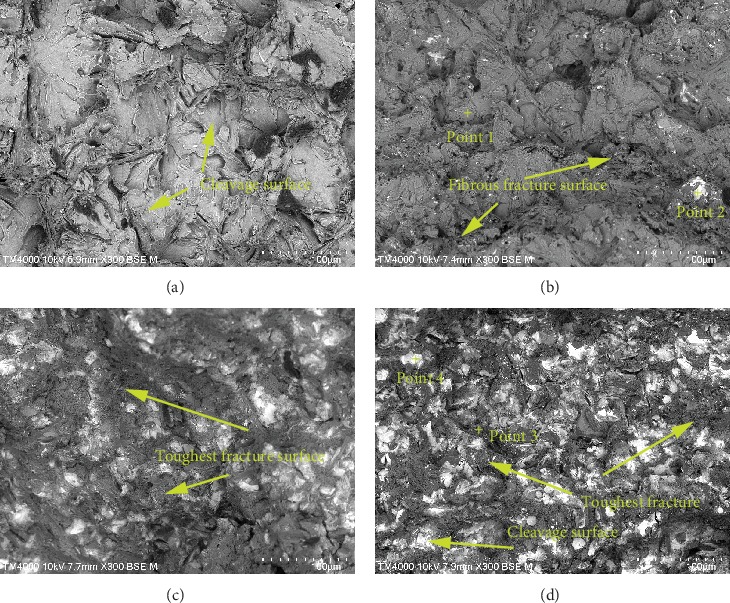
Fracture patterns of the Al-10Si-0.8Fe alloy with different amounts of Er: (a) unmodified, (b) 0.50 wt.%, (c) 0.80 wt.%, and (d) 1.00 wt.%.

**Figure 5:**
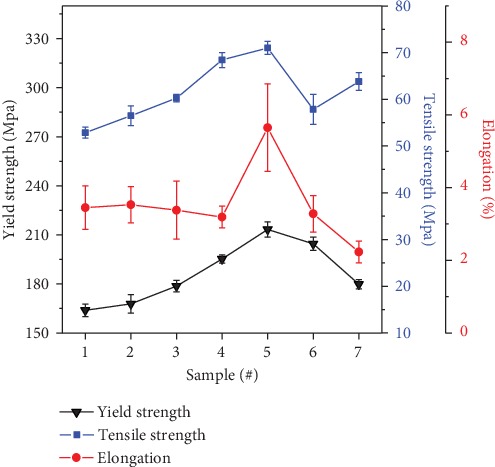
The YS, UTS, and %EL of the Al-10Si-0.8Fe alloy as functions of Er concentration.

The micrographs of the Al-10Si-0.8Fe-XEr alloy are shown in Figure 4.  Figure 4(a)) shows the SEM image without Er, and it is clearly seen that the fracture surfaces are mainly covered by a cleavage surface without Er. This is due to transgranular failure under the action of normal stress. The tensile fracture of the alloy showed a brittle fracture. It can be clearly seen from Figure 4(b) that the cleavage platform becomes smaller and a small number of dimples are formed. A small amount of white phase appears at the same time. Fractures also appear on the fibrous fracture surface. In addition, the number of folds and dimples increased with the increase of Er content. Meanwhile, seen from Figure 4(c), when the Er content reaches 0.8 wt.%, there are plenty of toughest fracture surface. And the white Er-rich phase is evenly distributed on the fracture surface, resulting in the best grain refining effect. Moreover, it can be observed that there are plenty of dimples present on the fracture surface. It is confirmed that the fracture mode of the alloy with the addition of 0.8 wt.% Er shows a mixed mode of brittle fracture and ductile fracture. However, when the addition of Er increased to 1.0 wt.%, a lager Er-rich phase appeared at fracture surface. It may reduce the tensile strength of the alloy.

In addition, EDS point analysis based on SEM (Figure 4) is shown at Table 3. According to Table 3, the gray cleaved surface (point 1) in Figure 4(b)) is the Al-Si phase. Point 2 is the white massive phase on the fracture. According to EDX, it can be known that the white phase is the Al-Si-Fe phase. And some Er elements are aggregated on the white phase. The fracture morphology corresponding to point 3 is gray fibrous. According to the results of EDX analysis, it can be known that the Er element content is higher here, which indicates that the Er element reacts with the matrix to form Er_2_Si, which refines the eutectic silicon, thereby improving the alloy's strength. It can be seen from Figure 4(d)) that a number of white phase increases as the amount of Er added increases, and they are more uniformly distributed on the substrate. As can be seen from EDX point 4 in Table 3, there is a great quantity of Er in the white phase, which means that excessive Er will coarse eutectic silicon, thereby reducing the tensile strength and increasing the brittleness of these alloys.

## 4. Discussion

In this study, the rare earth element Er can better refine the eutectic silicon in the Al-Si-Fe alloy, and the size of the eutectic silicon can be reduced by 38% when added in an appropriate amount. This is because during the nucleation and growth of *α*-Al grains during solidification, some Si and Fe atoms will enter the grains, and at the same time, some other elements will enrich the surface, making the *α*-Al grain size smaller [22]. The Er element added at the same time will generate the Er_2_Si phase which can be used as heterogeneous nuclei to promote grain refinement. The growth surface of the eutectic silicon is (111), and it is preferentially grown during the solidification and crystallization process. When no refiner is added, the eutectic silicon continuously grows to the sides by atomic deposition on the inherent step of the grain boundary, so that the appearance becomes coarse strip and block phase. After adding the rare earth element Er, it will be deposited on the grain boundary. This makes the growth of the silicon phase nonuniform and accumulates on the twins, resulting in constitutional supercooling. At the same time, the growth mode of silicon is changed, which changes the morphology of the Si phase [23]. The thermodynamic formula is as follows:

(3) $$\begin{array}{c} \Delta \text{Gv}=-\frac{\Delta H\Delta T}{\text{Tm}}, \end{array}$$

where *△*Gv is the phase change driving energy; △*H* is the latent heat of solidification; Tm is the equilibrium solidification temperature; and △T is the degree of subcooling.

According to the formula, when the subcooling degree becomes larger, the solidification driving force *Δ*Gv also becomes larger. The enrichment of Er element will hinder the growth of the Si phase and cause the component to be too cold, preventing the Si phase from growing. As a result, the morphology of the Si phase has changed from a bar shape to a short rod shape [24]. At the same time, some literatures pointed out that when Al and Si in the melt undergo eutectic transformation, a small amount of Er atoms are still present. And because Er atom has a smaller atomic radius and a larger atomic weight, it can be more easily enriched on the surface of eutectic grains. This greatly hindered the directional growth of eutectic silicon, resulting in grain refinement [25, 26]. Obviously, according to the experimental results, the aluminum alloy grains are refined. We believe that maybe there are some strengthened mechanisms in the composites, which we have not considered.

## 5. Conclusions

1. In Al-10Si-1.5Fe-*X*Er alloys, Er can significantly modify the second phases (include primary silicon, eutectic silicon, and iron-rich phases) of the Al-10Si-0.8Fe alloy that the morphology transfers from the coarse needle to fine granular and its size decreases to 1.87 *μ*m when the addition of Er is 0.8%. However, a further increase in the amount of addition of Er more than 0.8% leads to coarsening of the second phases
2. Adding the Er element can improve the mechanical properties of the Al-Si-Fe alloy. Compare with the Al-10Si-1.5Fe alloy, when the Er addition is 0.8 wt. %, the YS, UTS, and %EL increase from 52.86 MPa, 163.84 MPa, and 3.45% to 71.01 MPa, 163.84 MPa, and 5.65%, respectively. However, excess Er can lead to a decrease in the strength and toughness of these alloys
3. Adding an appropriate amount of element Er to the iron-rich Al-Si alloy can precipitate the Er_2_Si phase in the structure and finely refine the eutectic silicon phase in the alloy structure

## Figures and Tables

**Table 1:** Summary of the experimental alloys with different Er addition (unit: wt.%).

Sample no.	Addition content	Chemical composition of the experimental alloys			
		Si	Fe	Er	Al
#1	0	10.00	0.80	0.00	Bal.
#2	0.20	10.12	0.78	0.18	Bal.
#3	0.50	9.96	0.75	0.47	Bal.
#4	0.65	10.31	0.76	0.65	Bal.
#5	0.80	9.86	0.72	0.82	Bal.
#6	1.00	9.77	0.78	1.04	Bal.
#7	1.50	9.98	0.83	1.48	Bal.

**Table 2:** Elemental contents determined by EDX for the locations marked in Figure 4 (unit: at.%).

Point no.	Atomic (Al)%	Atomic (Si)%	Atomic (Fe)%	Atomic (Er)%
1	65.18 ± 2.10	34.82 ± 1.07	—	—
2	38.25 ± 1.12	36.15 ± 1.15	25.60 ± 1.13	—
3	8.82 ± 0.15	51.65 ± 2.20	4.92 ± 0.11	34.61 ± 1.07
4	37.02 ± 1.08	14.17 ± 0.21	—	48.81 ± 2.25

**Table 3:** Elemental contents determined by EDX for the locations marked in Figure 4.

Point no.	Atomic (Al)%	Atomic (Si)%	Atomic (Fe)%	Atomic (Er)%
1	64.94 ± 1.10	35.06 ± 1.07	—	—
2	32.75 ± 0.74	33.82 ± 0.60	30.48 ± 1.20	2.95 ± 0.10
3	58.18 ± 1.42	4.77 ± 0.13	3.70 ± 0.07	33.35 ± 2.10
4	13.33 ± 0.10	3.78 ± 0.40	5.05 ± 0.30	77.84 ± 1.10

## Data Availability

The [Manuscript.doc] data used to support the findings of this study have been deposited in the [Investigation on Microstructures and Mechanical Properties of the Hypoeutectic Al-10Si-0.8Fe-xEr Alloy] repository (9147871). The datasets used or analyzed during the current study are available from the corresponding author on reasonable request. Actually, all data generated or analyzed during this study are included in this published article.
